# Cardiogenic Shock Clinical Presentation, Management, and In-Hospital Outcomes in Patients Admitted to the Acute Cardiac Care Unit of a Tertiary Hospital: Does Gender Play a Role?

**DOI:** 10.3390/jcm9103117

**Published:** 2020-09-27

**Authors:** Adrian Jerónimo, Marcos Ferrández-Escarabajal, Carlos Ferrera, Francisco J. Noriega, Jesús Diz-Díaz, Rodrigo Fernández-Jiménez, Angela McInerney, Antonio Fernández-Ortiz, Ana Viana-Tejedor

**Affiliations:** 1 Acute Cardiac Care Unit, Hospital Clínico San Carlos, 28040 Madrid, Spain; adrijeronimo@gmail.com (A.J.); marcos_ferres@hotmail.com (M.F.-E.); carlosferreraduran@gmail.com (C.F.); noriegafj@hotmail.com (F.J.N.); jcarlavilla@hotmail.com (J.D.-D.); rodrigo.fernandez@cnic.es (R.F.-J.); angela_mcinerney@hotmail.com (A.M.); antonio.fernandezortiz@salud.madrid.org (A.F.-O.); 2 Centro Nacional de Investigación Cardiovascular (CNIC), 28029 Madrid, Spain

**Keywords:** cardiogenic shock, myocardial infarction, gender

## Abstract

Cardiogenic shock (CS), as the most severe form of heart failure, is associated with very high mortality rates despite therapeutic advances in the last decades. Gender differences in outcomes have been widely reported regarding several cardiovascular diseases. The aim of our study was to evaluate potential gender disparities in clinical presentation, management, and in-hospital outcomes of all (*n* = 138) patients admitted to the Acute Cardiac Care Unit of a tertiary hospital from 2013 to 2019. Information on demographic characteristics, past medical history, haemodynamic and clinical status at admission, therapeutic management, and in-hospital outcomes was retrospectively collected. Women represented 31.88% of the cohort, were significantly older than the men and had a lower proportion of smokers, chronic obstructive pulmonary disease, and previous acute myocardial infarction (AMI). Most CSs in both groups were AMI-related. Left ventricular ejection fraction at admission was higher in women, who were less likely to receive vasopressors. No differences were observed regarding mechanical circulatory support use and in-patient outcomes, with age being the only factor associated with in-hospital mortality on multivariate analysis.

## 1. Introduction

Cardiogenic shock (CS) is an extremely serious clinical condition. Management of CS remains challenging despite advances in therapeutic options [1,2]. Acute myocardial infarction (AMI) and subsequent ventricular failure is the most frequent cause of CS, representing more than 80% of cases [3]. Widespread implementation of early revascularization has achieved a significant reduction in mortality rates by up to 40–50%. However, CS is still associated with a very high mortality risk in AMI patients [4,5,6,7]. On the other hand, non-AMI-related CS may be secondary to several cardiac conditions, such as decompensated chronic heart failure, valvular heart disease, acute myocarditis, or Takotsubo syndrome. Revascularization therefore has little impact on mortality in this subgroup of patients [2,3]. 

Significant gender differences in clinical presentation, management, and prognosis of several cardiovascular diseases have been extensively reported [8]. This is especially true in AMI, where women are less likely to receive guideline-based pharmacological therapies and prompt revascularization than men, leading to higher mortality rates [9]. However, limited information on gender disparities in clinical characteristics, management, and outcomes of patients with CS has been reported [10].

The aim of our study was to evaluate potential gender disparities in the clinical presentation, management, and in-hospital outcomes in patients with CS admitted to the Acute Cardiac Care Unit (ACCU) of a tertiary referral hospital.

## 2. Material and Methods

This is a retrospective cohort study. All consecutive patients with CS admitted to the ACCU of a tertiary hospital from January 2013 to December 2019 were included. CS was defined, according to the European Society of Cardiology (ESC) guidelines [11], as systolic blood pressure < 90 mmHg without adequate response to volume replacement or need for vasopressor therapy to maintain systolic blood pressure > 90 mmHg; clinical signs of hypoperfusion (cold extremities, oliguria, mental confusion, dizziness); or consistent laboratory results (metabolic acidosis, elevated serum lactate, elevated serum creatinine). Those patients with CS secondary to cardiac surgery were not considered for this study.

When coronary-disease-related causes were presumed based on clinical presentation, or when electrocardiographic or echocardiographic findings suggested myocardial ischemia, a prompt coronary angiogram was performed. Immediate percutaneous treatment of the culprit lesion was attempted in all cases when technically feasible. Management of non-culprit lesions (complete or staged revascularization) was conducted according to the standard of care at the time of the study. 

Vasopressors and inotropic agents were initiated in cases of persistent hypotension with signs of hypoperfusion despite adequate filling status in order to maintain vital organ perfusion. According to local protocols, mechanical circulatory support devices were indicated in cases of cardiogenic shock refractory to vasoactive drugs, depending on the patient’s age, comorbidities, the possibility of recovery, or further therapies. Device choice was at the discretion of the multidisciplinary team including a cardiologist specializing in advanced heart failure, a specialist in acute cardiac care, an interventional cardiologist, and a cardiac surgeon.

Renal replacement therapy was indicated in patients with acute kidney injury and refractory volume overload who failed to respond to diuretics, hyperkalaemia, or severe metabolic acidosis. Non-invasive positive pressure ventilation was initiated in cases of respiratory failure leading to hypoxemia (PaO_2_ < 60 mmHg), hypercapnia (PaCO_2_ > 45 mmHg), acidosis (pH < 7.35 and >7.25), or clinical findings of tachypnoea with accessory respiratory muscles use, maintaining an adequate level of consciousness. Following local protocols, in cases of more severe respiratory failure or progressive deterioration despite non-invasive mechanical ventilation or intubation with mechanical ventilation were indicated.

The study was approved by the Local Ethical Committee (20/577-E). Information related to patients’ demographic characteristics and clinical history was collected. Data related to the index episode of CS were also recorded, including the underlying cause, the haemodynamic status at admission, left ventricle ejection fraction (LVEF), treatments administered, and clinical outcomes.

### Statistical Analysis

Quantitative variables are reported as mean and standard deviation and were compared with Student’s *t*-test. Categorical variables are expressed as percentages. Differences among them were assessed with a chi-square test or Fisher’s exact test as appropriate.

Variables in which statistically significant differences were observed in the univariate model and those clinically relevant were introduced into a multivariate analysis to identify those factors independently associated with mortality in the context of CS.

All tests were two-sided, and differences were considered statistically significant at *p*-values <0.05. Statistical analyses were performed with Stata/IC12.1 (StataCorp, College Station, TX, USA).

## 3. Results

### 3.1. Baseline Characteristics

A total of 138 patients were included. Women represented 31.88% of the study cohort and were significantly older than men (mean age 76.0 vs. 70.8, *p* = 0.019). Baseline characteristics of the study population are shown in Table 1.

A significantly lower proportion of smokers, numerically less chronic obstructive pulmonary disease (COPD), and a higher prevalence of hypertension were found in the female group. However, no differences were observed in the distribution of other cardiovascular risk factors, such as dyslipidaemia, diabetes, chronic kidney disease, or body mass index (BMI) (Table 1).

Regarding cardiovascular history, a previous AMI was documented in 9.30% of women and 28.72% of men (*p* = 0.012). However, coronary revascularization, regardless of the indication, had been performed in only 11.63% of women (80% percutaneously and 20% surgically) compared with 28.73% of men (85.19% percutaneously and 14.81% surgically) (*p* = 0.088). There were no differences regarding previous history of heart failure, stroke, or peripheral arteriopathy between both groups (Table 1).

### 3.2. Clinical Presentation

**Figure 1:**
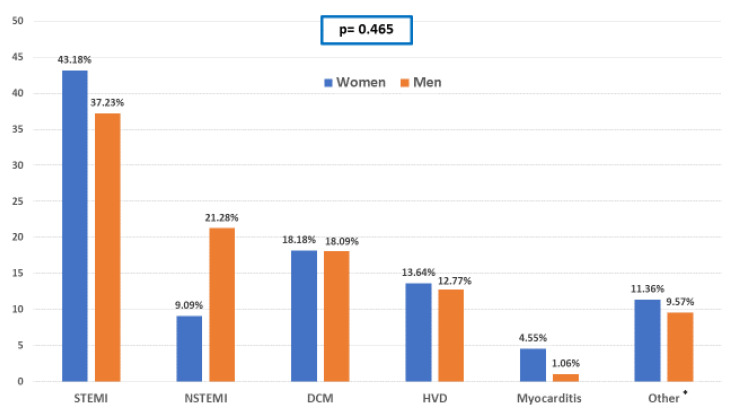
Causes of cardiogenic shock (CS). DCM: dilated cardiomyopathy; HVD: heart valve disease; NSTEMI: non-ST-segment elevation myocardial infarction; STEMI: ST-segment elevation myocardial infarction. * Other causes of CS included two cases of pulmonary embolism, one case of Takotsubo Syndrome, one case of tachycardiomyopathy, and nine cases of arrhythmic storm/sustained ventricular tachycardia. Statistical comparisons: Chi-square test, Fisher’s exact test.

ST-segment elevation myocardial infarction (STEMI) and non-ST-segment elevation myocardial infarction (NSTEMI) were the underlying causes of CS, in 29.13% and 17.39% of the patients, respectively. Mechanical complications of MI were documented in one female and three male patients. Causes of CS are summarized in Figure 1. No significant differences were observed in the underlying causes of CS between both groups.

Regarding clinical presentation, patients’ status at admission was similar in both groups (Table 2). Initial LVEF documented in women was significantly higher than in men (33.57% vs. 28.42%; *p* = 0.037). However, no statistically significant differences were observed regarding systolic and diastolic blood pressure, heart rate, lactate levels, or renal function parameters at admission. The proportion of sudden cardiac arrest and length of stay did not differ according to gender (Table 2).

### 3.3. Therapeutic Management

Concerning vasopressor support, dobutamine, noradrenaline, and levosimendan were used significantly less often in women than in men (Table 3). However, no significant differences were observed regarding the use of dopamine and adrenaline. Likewise, we found no differences between the need for invasive and non-invasive mechanical ventilation, renal replacement therapies, or Swan Ganz catheter placement between groups (Table 3).

With respect to mechanical circulatory support, 68.18% of women and 59.57% of men were treated with cardiac assist devices (*p* = 0.331). An intra-aortic balloon pump (IABP) was used in 29.55% of female and 37.23% of male patients (*p* = 0.377). Extra-corporeal membrane oxygenation (ECMO) was required in 2.27% of women and 4.26% of men (*p* = 0.561). Among male patients, five received support with Impella^®^, while no women were treated with an Impella® device (*p* = 0.119). Finally, one woman and two men underwent the implantation of a Levitronix Centrimag^®^ circulatory support system (*p* = 0.957) during the study period (Table 3).

**Figure 2:**
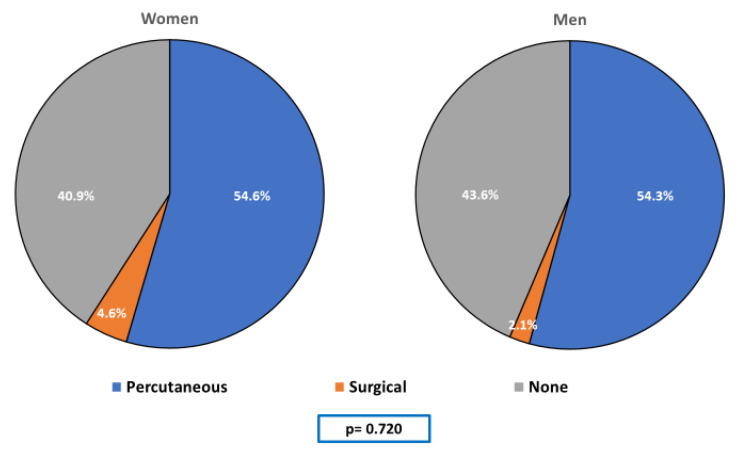
Revascularization approach according to gender. Statistical comparisons: Chi-square test, Fisher’s exact test.

Seventy-five (54.35%) patients included in the study underwent percutaneous revascularization, whereas four (2.90%) were treated surgically, without any significant differences between both groups (Figure 2). A total of 16.67% of women and 15.56% of men persisted with thrombolysis in myocardial infarction (TIMI) coronary flow grade 2 or worse after revascularization (*p* = 0.904).

### 3.4. In-Hospital Prognosis

In-hospital mortality rate was 47.73% in the female group and 47.87% in the male group (*p* = 0.987). Cardiovascular causes of death accounted for 85.71% in women and 79.55% in men (*p* = 0.549). Among the non-cardiovascular causes of death in women, one died from anoxic encephalopathy, another from sepsis, and another from mixed (distributive/cardiogenic) shock. Within the male group, four patients died from sepsis and another seven from mixed shock.

### 3.5. AMI-Related Episodes of CS

As AMI represents the most frequent cause of CS, we analyzed infarct-related cases in detail. Among these patients (n = 78), women represented 29.48%, being significantly older than men and having a greater prevalence of hypertension. Smoking was less frequent amongst women, but no significant differences were observed regarding other cardiovascular risk factors, previous history of MI, prior revascularization, heart failure, peripheral arteriopathy, or stroke (Table S1).

STEMI and NSTEMI accounted for 82.61% and 17.39% of presentations in women, respectively, while in men, these proportions were 63.64% and 36.36% (*p* = 0.098).

No significant differences according to gender were observed regarding haemodynamic and clinical status at admission, including LVEF (*p* = 0.186) (Table S2). Dobutamine and levosimendan were utilized significantly less often in women (*p* = 0.001 and *p* = 0.021, respectively), but no other differences were identified in relation to the use of other vasopressors, mechanical circulatory support, and invasive procedures (Table S3).

With respect to in-hospital mortality, no significant differences were observed according to gender (47.83% in women and 52.73% in men, *p* = 0.693). Moreover, cardiovascular causes represented over 90% of the total number of deaths in AMI-related cases (*p* = 0.837) (Table S3).

### 3.6. Non-AMI-Related Episodes of CS

Cases of non-AMI-related CS (n = 60) were mainly secondary to dilated cardiomyopathy, without any significant differences regarding the underlying causes of the episodes. Compared to women, men presented more frequently with chronic kidney disease (*p* = 0.042), previous MI (*p* = 0.013), and previous revascularization (*p* = 0.020). No other gender disparities were observed including regarding age, cardiovascular risk factors (except for a greater proportion of smokers among men), other comorbidities, or haemodynamic status at admission (Tables S1 and S2).

Regarding therapeutic management, women less frequently received vasoactive support with dobutamine (*p* = 0.051) and noradrenaline (*p* = 0.028), whereas the proportion of patients undergoing mechanical circulatory support was similar in both groups (Table S3). No gender disparities were found regarding in-hospital mortality and causes of death within this subgroup of patients.

### 3.7. Multivariate Analysis

A multivariate analysis was conducted to determine which factors may be associated with mortality in CS. Age and lactate levels at admission were found to be independently associated with in-hospital mortality in our cohort (Table 4). Gender was not found to be an independent predictor of in-hospital mortality.

## 4. Discussion

The present study has the following main findings: (1) almost one-third of patients admitted at the ACCU were women; (2) they were significantly older than men and less frequently smokers; (3) the proportion of women with a previous AMI was lower, but no differences among the causes of CS were observed according to gender status, with AMI-related cases occurring in over 50% in both groups; (4) LVEF at admission was higher in women than men, and they less frequently received vasopressor therapy; (5) no differences were observed in mechanical circulatory support use; (6) in-hospital mortality rate and causes of death were similar in both groups; and (7) age and lactate levels at admission were independently associated with in-hospital mortality, but gender wdid not predict in-hospital mortality.

Cardiovascular disease remains the leading cause of death worldwide across both genders [12]. Compared to men, women with AMI have a higher unadjusted mortality rate and lower use of guideline-recommended therapies, such as early intervention, reperfusion therapy, administration of thienopyridines, and glycoprotein inhibitors [13,14,15]. Moreover, gender-related management disparities have also been reported in the context of chronic heart failure [16,17,18] and cardiogenic shock [10,19].

Although the incidence of CS, especially complicating AMI, has been reported to be higher in women [20,21], the proportion of women in our study was 31.88%, consistent with recent reports by Collado-Lledó et al. (28%) [22], Fengler et al. (31%) [19], and Kunadian et al. (29.5%) [23], among others.

Consistent with our cohort, previous studies have also found women presenting with CS to be significantly older than men [12,16,17,18,19,22], however, the mean age of both groups in our study (76 years in women and 70.8 in men) were much higher than those already reported. Collado-Lledó et al. reported mean ages of 67 and 64 years old for women and men, respectively [22]. Similiarly, Kunadian et al. described mean ages of 69.9 and 64.2 years old for the female and the male groups, respectively [23]. The age difference between our study and those others mentioned may be attributed to the presence of heart transplantation and advanced heart failure programs in the centres participating in the studies of Collado-Lledó and Kunadian which result in a bias towards younger patients considered suitable for transplant.

Consistent with most studies, women were less likely to be smokers, less likely to have COPD, and less likely to have had previous AMI [12,16,17,18,19,22]. A tendency to a higher percentage of hypertension was also observed among women, which reached statistical significance among AMI-related cases. No differences were observed regarding other cardiovascular risk factors, heart failure, stroke, or peripheral vascular disease. However, in the AMI-related CS group, women had a higher cardiovascular risk profile in comparison with men (47.83% of diabetes vs. 40.00%; 52.17% of dyslipidaemia vs. 47.27%; and mean BMI of 27.27 vs. 25.87) without reaching statistical significance. While a clear numerical tendency towards a higher risk factor profile is evident, failure to attain statistical significance may be due to our small sample size.

In contrast to most previously published series [10,12,19,20,21,22,23,24], we collected both AMI and non-AMI-related episodes of CS. STEMI and NSTEMI represented the main causes, accounting for more than 50% of the cases of CS in both groups. Although CS secondary to NSTEMI has been previously described as occurring more frequently in women [25], the proportion of NSTEMI related CS among women in our cohort was slightly lower than in the male group. Collado-Lledó et al. also recently reported a lower prevalence of AMI-related CS in women [22]. Amongst other causes of CS, no gender differences were observed. 

Regarding clinical presentation, no differences were observed in the haemodynamic parameters at admission (systolic blood pressure, *p* = 0.576; diastolic blood pressure, *p* = 0.390 or heart rate, *p* = 0.824). Additionally, lactate and creatinine levels, as well as estimated glomerular filtrate rate at admission, were similar in both groups. Notwithstanding this finding, and similar to previous reports in the field [12,22], LVEF in women was found to be significantly higher (*p* = 0.037).

Concerning therapeutic management, a significantly lower proportion of women receiving dobutamine, noradrenaline, and levosimendan was found. It is possible that our small sample size resulted in inadequate power to detect differences regarding any other clinical or hemodynamic parameters that could in part justify this discrepancy, apart from LVEF which was found to be higher in women and may explain the decreased need for inotropic and vasopressor support. No differences were documented in the proportion of invasive and non-invasive mechanical ventilation and renal replacement therapies. Likewise, mechanical circulatory support, in all of its modalities, was used in a similar proportion in both genders, in spite of the fact that some series have described a greater hemodynamic benefit of these therapies in women, especially when initiated prior to percutaneous coronary interventions [12]. Similarly, Collado-Lledó et al. reported a lower use of dobutamine among women, without significant differences regarding invasive ventilation, renal replacement therapy, therapeutic hypothermia, or mechanical circulatory support [22]. In our study, pulmonary artery catheter use was slightly higher in men, though no significant differences were observed (9.09% vs. 3.19%, *p* = 0.141), as it has been previously described [12,22].

With respect to in-hospital mortality, we found no significant differences between both groups (47.73% vs. 47.87%, *p* = 0.987), consistent with several other studies [10,12,19,23,24] and despite the lower use of inotropic and vasopressor agents. Regarding the causes of death, refractory CS was the main cause in both groups, although men showed a tendency to a higher proportion of deaths related to other causes, such as sepsis or mixed distributive shock. After performing a multivariable analysis, the patient’s age and lactate levels at admission were the only variables associated with in-hospital mortality in our cohort. The comparable outcomes between men and women in our cohort despite less frequent use of vasoactive agents in women may be potentially explained by a less severe disease course in women although confirmation of this hypothesis would require a larger cohort and should be the focus of further investigation.

Lastly, implementation of standardized STEMI management protocols have resulted in a significant reduction in therapeutic disparities according to gender. Long-term follow-up will be required to assess the impact of standardized care on longer-term clinical outcomes [26]. Our study was conducted in a single centre, whose locally agreed protocols are implemented regardless of patient gender or attending physician. This fact may have influenced our results, minimizing heterogeneity related to gender.

Several limitations to this study must be taken into account: (1) being an observational study, determination of causality may be restricted and confounding factors cannot be fully excluded; (2) due to the fact that it is a single-centre study, the number of patients included is relatively small and only allowed subgroup analysis according to AMI and non-AMI related causes of CS; (3) vasoactive inotropic score to better characterize clinical status was not available; (4) invasive hemodynamic monitoring with a Swan Ganz catheter was performed in a small proportion of the patients; (5) data regarding revascularization times in those cases of AMI-related CS were not collected; and (6) long-term outcomes of patients who were discharged were not assessed.

## 5. Conclusions

Women represented approximately one-third of cases of CS, were significantly older than men, and less often had a history of AMI.

No significant differences were observed regarding the underlying causes of CS, hemodynamic status at admission, in-hospital mortality, and causes of death according to gender. However, women presented with higher LVEF at admission and received vasopressors less frequently than men. The patient’s age at admission was the only variable associated with in-hospital mortality.

## Figures and Tables

**Table 1:** Baseline characteristics of the study cohort according to gender status.

	Women (*n* = 44)	Men (*n* = 94)	*p* Value
Age (years)	75.96 (11.33)	70.81 (12.09)	0.019
Smoking history	13 (29.55%)	61 (64.89%)	0.001
Hypertension	34 (77.27%)	58 (61.70%)	0.071
Dyslipidaemia	23 (52.27%)	49 (52.13%)	0.987
Diabetes	16 (36.36%)	39 (41.49%)	0.567
BMI	26.63 (0.759)	25.91 (0.373)	0.341
COPD	1 (2.27%)	11 (11.70%)	0.067
CKD	6 (13.64%)	21 (22.34%)	0.230
Previous MI	4 (9.30%)	27 (28.72%)	0.012
Heart failure	8 (19.05%)	29 (30.85%)	0.153
Stroke	3 (6.82%)	5 (5.32%)	0.725
Peripheral arteriopathy	2 (4.55%)	13 (13.83%)	0.102

BMI: body mass index; CKD: chronic kidney disease; COPD: chronic obstructive pulmonary disease; MI: myocardial infarction. Statistical comparisons: Chi-square test, Fisher’s exact test, Student’s *t*-test.

**Table 2:** Haemodynamic and clinical parameters at admission, management, and outcomes of CS episodes according to gender status.

	Women	Men	*p* Value
Mental confusion	47.73%	47.87%	0.987
Cold distal extremities	70.45%	68.09%	0.779
Systolic blood pressure at admission (mmHg)	88.84	91.16	0.576
Diastolic blood pressure at admission (mmHg)	54.66	52.20	0.390
Heart rate at admission (bpm)	92.02	91.02	0.824
Lactate levels at admission (mmol/L)	5.95	5.48	0.539
Creatinine levels at admission (µmol/L)	145.32	165.45	0.285
Estimated glomerular filtrate rate at admission (mL/min)	39.57	46.85	0.156
Acute kidney disease at admission	81.8	78.7	0.674
LVEF	33.57%	28.42%	0.037
Sudden cardiac arrest during hospitalization	24.47%	15.91%	0.256
Length of stay (days)	15.23	13.86	0.851

CS: cardiogenic shock; LVEF: left ventricle ejection fraction. Statistical comparisons: Chi-square test, Fisher’s exact test, Student’s *t*-test.

**Table 3:** Therapeutic management of CS episodes according to gender status.

	Women	Men	*p* Value
Dobutamine	38.64%	74.47%	0.001
Noradrenaline	88.64%	97.87%	0.021
Levosimendan	6.82%	22.34%	0.025
Adrenaline	9.09%	14.89%	0.346
Dopamine	22.73%	12.77%	0.136
Invasive mechanical ventilation	47.73%	56.38%	0.342
Non-invasive mechanical ventilation	13.64%	12.77%	0.887
Renal replacement therapy	11.36%	20.21%	0.201
Swan Ganz catheter placement	9.09%	3.19%	0.141
Any mechanical circulatory support	68.18%	59.57%	0.331
IABP	29.55%	37.23%	0.377
ECMO	2.27%	4.26%	0.561
Impella^®^	0.00%	5.32%	0.119
Levitronix Centrimag^®^	2.27%	2.13%	0.957
In-hospital mortality rate	47.73%	47.87%	0.987
Cardiovascular cause of death	85.71%	79.55%	0.549

ECMO: extra-corporeal membrane oxygenation; IABP: intra-aortic balloon pump. Statistical comparisons: Chi-square test, Fisher’s exact test.

**Table 4:** Multivariate analysis for in-hospital mortality of all patients in the study.

	Odds Ratio	95% Confidence Interval	*p* Value
Gender (male)	1.11	0.44–2.83	0.814
Age *	1.06	1.03–1.11	0.001
Previous MI	0.81	0.31–2.10	0.660
Dobutamine use	1.80	0.74–4.38	0.196
Noradrenaline use	4.09	0.40–41.73	0.235
LEVF **	0.99	0.96–1.02	0.480
Lactate levels at admission (mmol/L) ***	1.18	1.07–1.30	0.001

LEVF: left ventricle ejection fraction; MI: myocardial infarction. * For every increase in one year. ** For every increase in 1%. *** For every 1 mmol/L increase.

## Supplementary Materials

The following are available online at https://www.mdpi.com/2077-0383/9/10/3117/s1, Table S1: Baseline characteristics of the study cohort according to gender in AMI and non-AMI-related episodes of CS, Table S2: Haemodynamic and clinical parameters at admission, management and outcomes of CS episodes according to gender status in AMI and non-AMI-related episodes of CS, Table S3: Therapeutic management of CS episodes according to gender status in AMI and non-AMI-related episodes of CS.
