# Supplementary material for: Cardiogenic Shock Clinical Presentation, Management, and In-Hospital Outcomes in Patients Admitted to the Acute Cardiac Care Unit of a Tertiary Hospital: Does Gender Play a Role?
Source: J Clin Med. 2020 Sep 27;9(10):3117. doi: 10.3390/jcm9103117 (PMC7601399; doi:10.3390/jcm9103117)
Supplement: Supplementary file 1 [file jcm-09-03117-s001.pdf]

Table S1.- Baseline characteristics of the study cohort according to gender in AMI and non-AMI-related episodes of CS.

	**AMI-related CS**			**Non-AMI-related CS**		
	**Women** (n=23)	**Men** (n=55)	**p Value**	**Women** (n=21)	**Men** (n=39)	**p Value**
Age (years)	78.16 (2.31)	71.09 (1.66)	0.020	73.56 (11.40)	70.42 (11.93)	0.326
Smoking history	30.43%	69.09%	0.002	28.57%	59.97%	0.025
Hypertension	91.30%	56.36%	0.003	61.90%	69.23%	0.566
Dyslipidaemia	52.17%	47.27%	0.693	52.38%	58.97%	0.623
Diabetes	47.83%	40.00%	0.524	23.81%	43.59%	0.129
BMI	27.27 (0.98)	25.87 (0.52)	0.174	25.90 (1.19)	25.98 (0.52)	0.945
COPD	4.35%	14.55%	0.199	0.00%	7.69%	0.192
CKD	17.39%	14.55%	0.751	9.52%	33.33%	0.042
Previous MI	13.64%	25.45%	0.259	4.76%	33.33%	0.013
Previous revascularization	21.74%	32.73%	0.332	0.00%	23.08%	0.020
Heart failure	8.70%	16.36%	0.375	31.58%	51.28%	0.157
Stroke	4.35%	3.64%	0.882	9.52%	7.69%	0.807
Peripheral arteriopathy	4.35%	16.36%	0.148	4.76%	10.26%	0.463

BMI: body mass index; CKD: chronic kidney disease; COPD: chronic pulmonary obstructive disease; MI: myocardial infarction

Statistical comparisons: Chi-square test, Fisher’s exact test, Student’s t-test.

Table S2.- Haemodynamic and clinical parameters at admission, management and outcomes of CS episodes according to gender status in AMI and non-AMI-related episodes of CS.

	**AMI-related CS**			**Non-AMI-related CS**		
	**Women**	**Men**	**p Value**	**Women**	**Men**	**p Value**
Mental confusion	56.52%	49.09%	0.549	38.10%	46.15%	0.548
Coldness of distal extremities	65.22%	69.09%	0.738	76.19%	66.67%	0.443
Systolic blood pressure at admission (mmHg)	92.00 (6.12)	90.77 (2.93)	0.832	85.38 (3.79)	91.77 (3.59)	0.260
Diastolic blood pressure at admission (mmHg)	52.48 (3.21)	51.61 (2.15)	0.822	57.05 (4.09)	52.97 (2.25)	0.345
Heart rate at admission (bpm)	85.43 (5.45)	89.40 (3.26)	0.520	99.24 (5.56)	93.31 (3.73)	0.366
Lactate levels at admission (mmol/L)	6.07 (0.93)	5.90 (0.61)	0.879	5.82 (0.53)	4.91 (0.68)	0.367
Creatinine levels at admission (µmol/L)	112.00 (9.06)	136.91 (9.12)	0.108	181.83 (21.02)	205.70 (23.16)	0.502
Estimated glomerular filtrate rate at admission (mL/min)	46.40 (4.58)	51.61 (4.35)	0.481	32.09 (4.70)	40.15 (4.10)	0.225
LVEF	32.96% (3.19)	28.72 (1.56)	0.186	34.24% (3.69)	28.00% (2.04)	0.112
Sudden cardiac arrest during hospitalization	17.39%	23.64%	0.542	14.29%	25.64%	0.309
Length of stay (days)	13.39 (3.45)	9.13 (7.18)	0.708	17.24 (6.57)	20.54 (4.82)	0.687

CS: cardiogenic shock; LVEF: left ventricle ejection fraction.

Statistical comparisons: Chi-square test, Fisher's exact test, Student's t-test.

Table S3.- Therapeutic management of CS episodes according to gender status in AMI and non-AMI-related episodes of CS.

	**AMI-related CS**			**Non-AMI-related CS**		
	**Women**	**Men**	**p Value**	**Women**	**Men**	**p Value**
Dobutamine	26.09%	72.73%	0.001	52.38%	76.92%	0.051
Noradrenaline	95.65%	98.18%	0.519	80.95%	97.44%	0.028
Levosimendan	0.00%	20.00%	0.021	14.29%	25.64%	0.309
Adrenaline	8.70%	7.27%	0.830	9.52%	25.64%	0.137
Dopamine	26.09%	14.55%	0.226	19.05%	10.26%	0.339
Invasive mechanical ventilation	56.52%	58.28%	0.892	38.10%	53.85%	0.244
Non-invasive mechanical ventilation	17.39%	9.09%	0.295	9.52%	17.95%	0.383
Renal replacement therapy	8.70%	12.73%	0.611	14.29%	30.77%	0.160
Swan Ganz catheter implantation	8.70%	3.64%	0.356	9.52%	2.56%	0.238
Any mechanical circulatory support	52.17%	52.73%	0.964	85.71%	69.23%	0.160
IABP	47.83%	43.64%	0.734	9.52%	28.21%	0.094
ECMO	0.00%	5.45%	0.253	4.76%	2.56%	0.651
Impella®	0.00%	5.45%	0.253	0.00%	5.13%	0.291
Levitronix Centrimag®	4.35%	1.82%	0.519	0.00%	2.56%	0.459
In-hospital mortality rate	47.83%	52.73%	0.693	47.62%	41.03%	0.623
Cardiovascular cause of death	90.91%	92.86%	0.837	80.00%	56.25%	0.216

ECMO: extra-corporeal membrane oxygenation; IABP: intra-aortic balloon pump.

Statistical comparisons: Chi-square test, Fisher's exact test.
